# Negative pressure wound therapy for high-risk wounds in lower extremity revascularization: study protocol for a randomized controlled trial

**DOI:** 10.1186/s13063-015-1026-1

**Published:** 2015-11-04

**Authors:** Patrick Murphy, Kevin Lee, Luc Dubois, Guy DeRose, Thomas Forbes, Adam Power

**Affiliations:** Division of General Surgery, Department of Surgery, Western University, 1151 Richmond Street, London, ON N6A 5A5 Canada; Division of Vascular Surgery, Department of Surgery, Western University, 800 Commissioners Road East, London, ON N6A 4G5 Canada; Division of Vascular Surgery, Department of Surgery, University of Toronto, 149 College Street, Toronto, ON M5T 1P5 Canada

**Keywords:** Surgical site infection, Vascular surgery, Negative pressure wound therapy, Prevena

## Abstract

**Background:**

Rates of surgical site infections (SSIs) following groin incision for femoral artery exposure are much higher than expected of a clean operation. The morbidity and mortality is high, particularly with the use of prosthetic grafts. The vascular surgery population is at an increased risk of SSIs related to peripheral vascular disease (PVD), diabetes, obesity, previous surgery and presence of tissue loss. Negative pressure wound therapy (NPWT) dressings have been used on primarily closed incisions to reduce surgical site infections in other surgical disciplines. We have not come across any randomized controlled trials to support the prophylactic use of negative pressure wound therapy in high-risk vascular patients undergoing lower limb revascularization.

**Methods/design:**

In this single-center, prospective randomized controlled trial, patients scheduled for a lower limb revascularization requiring open femoral artery exposure who are at a high risk (BMI > 30 kg/m^2^, previous femoral cutdown or Rutherford V or VI category for chronic limb ischemia) will be eligible for the study. A total of 108 groin incisions will be randomized to the use of a negative pressure wound device or standard adhesive gauze dressing. Patients will be followed in hospital and reassessed within the first 30 days postoperatively. The primary outcome is SSI within the first 30 days of surgery and will be determined using the intention-to-treat principle. Secondary outcomes include length of stay, emergency room visits, reoperation, amputation and mortality. A cost analysis will be performed.

**Discussion:**

The trial is expected to define the role of NPWT in SSI prophylaxis for lower limb revascularization in high-risk vascular patients. The results of the study will be used to inform current best practice for perioperative care and the minimization of SSIs.

**Trial registration:**

NCT02084017, March 2014

## Background

Surgical site infections (SSIs) at the groin incision following lower extremity revascularization procedures have been reported as up to 30 % [1–6]. This is significantly higher than expected in a surgical wound classified as clean, 2.1–3.3 % [7]. A number of risk factors within this population has been identified as risk factors for SSIs and include obesity, previous femoral cutdown and presence of critical limb ischemia [8, 9]. Significant morbidity associated with SSIs in lower extremity revascularization includes increased length of stay, sepsis, hospital readmissions, limb loss, and death [10, 11]. Further, the cost of vascular SSIs has been recently estimated to add US$10,000 to the cost of care per patient [11].

A variety of methods including topical antibiotics, antibiotic-impregnated grafts, and platelet-rich plasma have all been used with limited success [12]. Negative pressure wound therapy (NPWT) is a relatively new treatment modality that has also demonstrated some evidence in SSI prevention in incisions closed primarily, including groin incisions [6, 13, 14]. However, there are currently no randomized controlled trials investigating the use of NPWT to prevent SSIs following lower limb revascularization.

The objective of this study is to assess the role of NPWT, using the Prevena Incision Management System (Kinetic Concepts Inc, San Antonio, TX, USA), in primary closed groin incisions in patients requiring femoral artery exposure for lower limb revascularization on decreasing SSI compared to standard sterile gauze dressing. We hypothesize that patients randomized to NPWT will have a lower incidence of SSI in the groin incision within the first 30 days of surgery compared to patients randomized to the control group.

## Methods/design

Western University ethics board performed a full-board review and approved the trial (REB 104871). The trial is registered at Clinicaltrials.gov (NCT02084017). The study will be funded by the Department of Surgery Resident Research grant from Western University. Kinetic Concepts Inc. (San Antonio, TX, USA) will donate all NPWT devices for this trial and will not have any influence in study design, data collection, statistical analysis or writing of the final manuscript.

### Study design

This will be a single institution, prospective, randomized, open-label trial, in which eligible patients will be randomized to NPWT or a standard dressing of groin incision using a block randomization.

### Study setting

The study will take place at the Victoria Hospital of London Health Sciences Center (LHSC) in London, ON, Canada: Victoria Hospital is an academic teaching hospital and is associated with the Schulich School of Medicine and Dentistry at Western University.

### Eligibility

High-risk patients scheduled for a lower limb revascularization requiring groin incision for a femoral artery exposure are eligible for the study. Example operations include but are not limited to femoral to femoral artery crossover bypass, femoral to distal artery bypass, and femoral artery endarterectomy. Before the enrolment, informed consent will be obtained from each patient. Primary author KL will identify and be responsible for enrolling all the eligible patients.

### Inclusion criteria

Patients must be 18 years or older and scheduled for a lower limb revascularization operation requiring femoral artery exposure. Groin incision must be closed primarily in the operating room. Patients with one of the high-risk factors: obesity (body mass index > 30 kg/m^2^), previous femoral artery exposure or presence of minor or major tissue loss, will be included in the study.

### Exclusion criteria

Patients who have a pre-existing groin infection, a known sensitivity/allergy to adhesive material or on whom a complete seal of NPWT cannot be obtained at the time of device application will be excluded.

### Intervention and control

The intervention group will have NPWT, the Prevena Incision Management System (Kinetic Concepts Inc, San Antonio, TX, USA), applied to the primary closed groin incision. The Prevena system has a silver interface with a foam layer connected to a small portable battery-operated vacuum unit. The vacuum is preset to continuous at 125 mmHg. The device is applied under sterile conditions in the operating room and remains on the patient until the hospital discharge or postoperative day 8, whichever occurs first. The wound will be inspected for any evidence of infection once the Prevena is removed. The device is used only once and is disposed of at the end of the study. Any concern about the wound or the device will require removal of the NPWT earlier than planned and will be recorded as a secondary endpoint of the trial.

The control group will have a standard sterile gauge dressing to cover the groin incision. The postoperative management is typically removal of the dressing on day 2 postoperatively with daily dressing changes thereafter. All other pre- and postoperative care of patients will be the same in both groups. Hair will be removed with electric clippers preoperatively. Skin preparation will be done with 2 % chlorhexidiene. Every patient will received preoperative antibiotics (cephalexin or vancomycin if penicillin allergy is present) and postoperative antibiotics will be given for 48 h. At the end of the operation, the groin incision will be primary closed with either skin staples, monocryl or prolene sutures.

### Randomization, allocation concealment and blinding

Once the groin incision is primary closed, randomization will be done using internet-based software, sealedenvelope.com (London, UK), via random permutated blocks. If the patient has bilateral groin incisions, the right groin will be randomized and the left groin will be automatically placed in the other arm. The surgical team, clinical staff, and patient will not be blinded to the intervention status. The primary outcome of SSI will be assessed by a clinical specialist nurse who will be blinded to the intervention status.

### Definition of primary endpoint and outcome measures

The primary endpoint is the incidence of superficial SSI within 30 days of surgery. SSI is defined by the Center for Disease Control and Prevention (CDC) criteria [15] as: infection occurring within the first 30 postoperative days involving skin and subcutaneous tissue of the incision and at least one of the following:

1. Purulent drainage from the incision
2. Organisms isolated from an aseptically obtained culture of fluid or tissue from the incision
3. At least one of the following signs/symptoms of infection
  Pain or tenderness
  Localized swelling
  Redness
  Heat
  OR incision is deliberately opened by a surgeon (unless incision is culture negative)

4. Diagnosis of SSI by the surgeon or attending physician

Once the SSI is identified, the Szilagyi classification of vascular wound infections will be used to classify to infection [16]. This classification categorizes vascular SSI based on dermal involvement (I), subcutaneous involvement (II) or arterial graft involvement (III).

SSI will be assessed daily, at discharge, and reassessed at the outpatient clinic within 30 days following the operation.

### Secondary endpoints

Secondary endpoints include length of stay, emergency room visits, SSI requiring surgical intervention, deep SSI occurring within 90 days of an operation, limb amputation, and mortality. A cost analysis will also be performed using the average cost provided by LHSC for inpatients, return visits, and any surgical intervention. Outcomes related to potential harm will also be identified including local reaction to the NPWT device, and the need for early removal of the dressing.

### Sample size

Our previous study revealed a SSI rate of 22 % in all patients requiring femoral artery exposure for vascular surgical procedure at LHSC [4]. This study will only include high-risk patients as defined above and we expect the wound infection rate to be higher. Matatov et al. demonstrated a reduction of groin SSI with NPWT from 30 % to 6 %, an absolute risk reduction of 24 % and relative risk reduction of 80 % [6]. Given an alpha of 0.05, for 80 % power, this yields a required sample size of 48 patients per group using the Fleiss method with continuity correction. The planned sample size will account for potential loss of follow-up, device malfunction and patient incompliance and has been adjusted to a final sample size of 54 (10 % increase from calculated sample size) groin incisions per group and a total study sample of 108 groin incisions. The timeline for recruitment is 20 months and with a 90-day follow-up the study will be concluded in 2 years.

### Data collection and management

All data collected will be recorded on paper forms and will be kept within the patient charts. Data will be collected by the surgical team and trial personnel. Accuracy of data collection will be ensured by the study personnel not involved with data management or analysis by performing sample assessments at regular intervals. Any adverse events due to NPWT that causes morbidity or mortality will be recorded and reported to the primary investigator and institutional ethics committee. We expect minimal to no harm from the use of NPWT in this trial based on a previous report using the same device [6].

### Statistical analysis

All analysis will be prespecified and conducted according to the intention-to-treat principle with the use of SPSS version 20 (IBM, Armonk, NY, USA). Each incision will be randomized to the NPWT or standard dressing group. In the case that a patient requires bilateral incisions, the right side will be randomized and the left side will be automatically placed in the opposite arm but not be included in the primary analysis as there is a loss of independence. Baseline characteristics of the two groups will be recorded. The continuous variables will be compared using Student’s *t* test. The categorical variables will be compared using Pearson’s chi-square or Fisher’s exact test depending on the number of events. The proportion of SSIs amongst randomized groins will be compared between the NPWT and standard dressing groups using a chi-square test or Fisher’s exact tests depending on the number of events. An absolute risk increase/reduction for SSI will be presented for the use of NPWT, as well as the number needed to treat to prevent a single SSI. Patients lost to follow-up have the outcome last assessed treated as his or her final outcome. A secondary analysis will be performed including the nonrandomized groin in patients with bilateral incisions. We will also perform a subgroup analysis on just patients undergoing bilateral incision using McNemar’s test.

Secondary outcomes will be compared between groups using a chi-square test for categorical variables. Non-normally distributed continuous variables will be compared using the Mann–Whitney *U* test. A *p* value of less than 0.05 will be considered significant and all tests will be two-sided.

## Discussion

This trial is the first randomized controlled trial to investigate the role of NPWT in reducing the incidence of SSIs following surgery for lower limb revascularization in high-risk patients. Matatov et al. retrospectively reviewed 115 groin incisions in 90 patients who received either NPWT or adhesive gauze dressing following vascular procedure [6]. The authors demonstrated a reduction of SSI from 30 % to 6 % and no grade II or III infections in the NPWT cohort [6]. Our hypothesis is that high-risk patients in this study will see a similar benefit of reduction of SSIs.

Draining fluid following lower limb revascularization is not a new concept and in the past surgical drains placed deep in the wound have been investigated with randomized trials and meta-analyses, which did not show a reduced rate of SSI [17–19]. Significant research has been invested into the mechanism of action of NPWT on wound healing leading to four primary effects: macrodeformation; stabilization of the wound environment; reduced edema and microdeformation [17]. In conjunction with reticulated open-cell foam, such as used in the current study, NPWT can have a stimulatory effect on cellular proliferation and angiogenesis. Further, by actively removing excessive interstitial fluid and edema, it is thought secondary necrosis is reduced [18]. On a molecular level, cytokine and growth factor expression are modulated to an anti-inflammatory profile and culminate in angiogenesis, remodeling and granulation tissue [19]. The closed suction environment placed under sterile conditions also effectively separates the incision from the surrounding environment theoretically preventing the inoculation of environmental bacteria.

This trial will clarify the role of NPWT and its potential for reduction in SSI in groin incisions for lower limb revascularization. The major limitation of this trial is the inability to blind the surgical team and the patient. We will randomize the incisions once the incision is primary closed to reduce the bias that could occur from the surgical team.

The results of this trial will be used to determine the role of NPWT in the prevention of SSI. If this intervention is shown to be effective for the prevention of SSI in this high-risk population, significant benefit with respect to both patient morbidity and resource utilization may be achieved.

## Trial status

Enrollment started on August 13, 2014. Currently, 46 patients have been enrolled.

## Abbreviations

CDC: Center for Disease Control and Prevention
LHSC: London Health Sciences Center
NPWT: negative pressure wound therapy
SSI: surgical site infection
